# Immunoexpression Pattern of Autophagy Markers in Developing and Postnatal Kidneys of *Dab1*^−/−^ *(yotari*) Mice

**DOI:** 10.3390/biom13030402

**Published:** 2023-02-21

**Authors:** Mirko Maglica, Nela Kelam, Ejazul Haque, Ilija Perutina, Anita Racetin, Natalija Filipović, Yu Katsuyama, Katarina Vukojević

**Affiliations:** 1 Department of Anatomy, School of Medicine, University of Mostar, 88000 Mostar, Bosnia and Herzegovina; 2 Department of Anatomy, Histology and Embryology, University of Split School of Medicine, 21000 Split, Croatia; 3 Department of Medical Genetics, School of Medicine, University of Mostar, 88000 Mostar, Bosnia and Herzegovina; 4 Department of Anatomy, Shiga University of Medical Science, Otsu 520-2192, Japan; 5 Center for Translational Research in Biomedicine, University of Split School of Medicine, 21000 Split, Croatia

**Keywords:** LC3B, GRP78, HSC70, LAMP2A, kidney development, *yotari*, immunofluorescence staining, CAKUT

## Abstract

The purpose of this study was to compare the immunofluorescence patterns of autophagic markers: Light chain 3 beta (LC3B), Glucose regulating protein 78 (GRP78), Heat shock cognate 71 (HSC70) and Lysosomal-associated membrane protein 2A (LAMP2A) in the developing and postnatal kidneys of *Dab1^−/−^* (*yotari*) mice to those of wild-type samples. Embryos were obtained on gestation days 13.5 and 15.5 (E13.5 and E15.5), and adult animals were sacrificed at postnatal days 4, 11 and 14 (P4, P11, and P14). After fixation and dehydration, paraffin-embedded kidney tissues were sectioned and incubated with specific antibodies. Using an immunofluorescence microscope, sections were analyzed. For statistical analysis, a two-way ANOVA test and a Tukey’s multiple comparison test were performed with a probability level of *p* < 0.05. A significant increase in GRP78 and LAMP2A expression was observed in the renal vesicles and convoluted tubules of *yotari* in embryonic stages. In postnatal kidneys, all observed proteins showed higher signal intensities in proximal and distal convoluted tubules of *yotari*, while a higher percentage of LC3B-positive cells was also observed in glomeruli. Our findings suggest that all of the examined autophagic markers play an important role in normal kidney development, as well as the potential importance of these proteins in renal pathology, where they primarily serve a protective function and thus may be used as diagnostic and therapeutic targets.

## 1. Introduction

Congenital anomalies of the kidney and urinary tract (CAKUT) are disorders that emerge during renal development and result in a spectrum of structural defects in kidneys and outflow tracts (ureters, bladder and urethra). Depending on the registry, these malformations occur in 4–60 per 10,000 children [1,2]. Kidney development is a multi-stage process that begins with the induction of the ureteric bud from the nephric duct, followed by mesenchymal-to-epithelial transition and branching morphogenesis and terminates with the completion of nephron patterning and elongation [3]. Interferences to normal nephrogenesis, due to exposure to environmental risk factors or the dysfunction of genes that control this process, can lead to CAKUT [4,5]. Using mouse models, we obtained most of the current knowledge regarding CAKUT and genes involved in renal development and nephrogenesis [6]. From minor defects to syndromic presentations affecting not just the genitourinary tract but also other fetal structures and amniotic fluid, CAKUT presents in a variety of ways and can be identified as early as 11 weeks of gestation in humans. Consequently, identifying the genes responsible for CAKUT can help physicians in early diagnostics, as well as in the therapy of these frequent anomalies [7]. The Disabed-1 (Dab1) and Reelin regulate neuronal migration during brain development. The binding of Reelin to its receptors induces Dab1 tyrosine phosphorylation. Tyrosine-phosphorylated Dab1 recruits a wide range of SH2 domain-containing proteins and activates multiple signaling cascades. Their signaling pathway has a well-established role in regulating the “inside-out” lamination of the cerebral cortex and the activation of intracellular signaling cascades and rearrangement of the cytoskeleton to guide neuronal migration [8,9]. Recently, our group demonstrated the appearance of DAB1 and REELIN during human fetal kidney development and confirmed their potentially significant role in early kidney nephrogenesis [10]. Additionally, we found that homozygous *Dab1^−/−^* mutation causes autosomal recessive CAKUT displayed with kidney hypoplasia [11]. Therefore, this study aims to look into the potential causes of kidney hypoplasia in *yotari* and the role of autophagy in kidney cell degradation via lysosome-dependent cell removal.

In contrast with apoptosis, autophagy maintains cellular homeostasis by recycling selective organelles and useful molecules. During autophagy, unique autophagosomes are formed and fused with lysosomes to form autolysosomes [12,13]. Light chain 3 (LC3) family member, LC3B, is recognized as an autophagy-related protein [14]. During the fusion of autophagosomes with lysosomes, intra-autophagosomal LC3B-II (a post-translation modification form of LC3B) is degraded by lysosomal proteases, which makes these proteins markers of autophagic activity [15,16]. It was shown to be down-regulated in kidneys after injury, suggesting that autophagy might be involved in repairing of kidney injuries [17].

A specific type of autophagy called chaperone-mediated autophagy (CMA) is important for eliminating oxidized proteins. The main characteristic of this mode is the presence of so-called lysosomal-associated membrane protein 2A (LAMP2A) [18,19]. A study from 2017 by Zhang et al. analyzed the distribution of LAMP2A in disease-relevant kidneys. When compared to wild-type mouse kidneys, their findings revealed an increase in the percentage of LAMP2A-positive cells apically but a lack of lysosomal proteins in the basal areas of proximal tubule cells, implying defective LAMP2A trafficking and thus inhibited CMA activity, potentially leading to progressive renal injury in cystinosis [20].

Chaperones, also called heat shock proteins, and their cofactors are important for regulating the apoptotic pathway. They facilitate the restoration of normal function by refolding denatured and degrading irreparably damaged proteins via autophagy [21]. Glucose-regulated protein 78 (GRP78) belongs to the Heat shock protein 70 (HSP70) family. Its expression is induced during endoplasmic reticulum stress and its main role is in preventing ER stress-induced cell death [22,23,24]. ER stress was observed in different kidney diseases, such as diabetic nephropathy, renal fibrosis, genetic mutations of renal proteins, proteinuria, idiopathic nephrotic syndrome, and minimal change renal disease [25].

Heat shock cognate 71 (HSC70) protein displays significant homology with HSP70 (around 75%). It is mostly found in the cytoplasm, where it has been demonstrated to support a variety of oncogenic processes by controlling client proteins [26]. To perform CMA, HSC70 binds unfolded/misfolded proteins with an exposed KFERQ (consensus peptide sequence found in various native proteins) amino acid motif and brings them to the lysosome. The HSC70/KFERQ-containing client protein complex then interacts with and binds to the cytosolic tail of the monomer form of the receptor for CMA, lysosome-associated membrane protein 2A (LAMP-2A) [27]. In a recent study, Zhang et al. demonstrated that kidney cancer cells, particularly those from patients with metastases, expressed HSC70 staining in the nucleus and/or cytoplasm in a significantly higher percentage than the kidneys of the control group of patients [28].

This study aimed to analyze the effects of *Dab1* gene functional silencing on the expression and localization of LC3B, LAMP2A, GRP78 and HSC70 in the developing and postnatal kidneys of *Dab1^−/−^* (*yotari*) mice. Understanding the normal expression of these autophagy markers can lead to better diagnostic and treatment modalities for kidney diseases.

## 2. Materials and Methods

### 2.1. Ethics

Animal use was approved by the Guidelines for the Care and Use of Laboratory Animals at the Shiga University of Medical Science. The study was conducted according to the guidelines of the Declaration of Helsinki and approved by the Ethical Committee of the University of Split School of Medicine (UP/1-322-01/17-01/13; 525-10/0255-17-7; 13 October 2017).

### 2.2. Generation of Dab1 Null Conventional Mutants and Sample Collection

The *yotari* (*Dab1^−/−^*) mouse is a neurological mutant mouse with a phenotype similar to a *reeler (Reelin^−/−^)* mouse. These mice arose unexpectedly in the descendants of a male chimeric mouse carrying a gene mutation encoding the receptor for inositol-1,4,5-trisphosphate (IP3R1) K.O. mice [29,30]. *Yotari* mouse, which spontaneously arose from a mutation in *Dab1,* and the *reeler* mouse, whose mutation is caused by deletion of the 3′ coding region of reelin cDNA, both exhibit the same phenotype pattern, including unstable gait, tremors, and early death around the time of weaning [31,32,33,34]. These similarities in the phenotypes of *yotari* and *reeler* mice suggest that the gene mutated in *yotari* encodes a molecule on the same signaling pathway as Reelin, the product of the *reelin* gene [29].

In a temperature-controlled (23 ± 2 °C) setting, *yotari* (*yot*) and C57BL/6N (wt) mice, colonies genetically identical within each strain, making them free of genetic differences that could impact research results [35], were grown and housed separately in groups of three to four in typical polycarbonate cages with free access to food and tap water. Three mice were used for each genotype (*yotari* and wt) at every observed time point. The photoperiod consisted of 12 h of artificial light and 12 h of darkness. The following PCR primers were used for genotyping: *yotari*—GCCCTTCAG-CATCACCATGCT and CAGTGAGTACATATTGTGTGAGTTCC, wild-type of *Dab1* locus—GCCCTTCAGCATCACCATGCT and CCTTGTTTCTTTGCTTTAAGGCTGT [36].

On gestation days 13.5 (E13.5) and 15.5 (E15.5), the gravid mice were sacrificed, and their embryos were obtained. Postnatal groups of mice were sacrificed on their 4th, 11th and 14th postnatal days (P4, P11, P14). With pentobarbital, mice were deeply anesthetized before being transcardially perfused with phosphate buffer saline (PBS, pH 7.2) followed by 4% paraformaldehyde (PFA) in 0.1 M PBS. Kidneys were removed and 4% PFA in 0.1 M PBS was used to fix them overnight for conventional histological analyses (hematoxylin-eosin immunofluorescence staining). All chemicals used were cerified by the Biological Stain Commision and were used as received without any further purification, and were obtained from Sigma-Aldrich (St. Louis, MO, USA)

### 2.3. Immunofluorescence

After fixation, tissue was dehydrated with graded ethanol solutions (Sigma-Aldrich, St. Louis, MO, USA), embedded in paraffin blocks and serially cut as 5 µm-thick sections, which were then mounted on glass slides. Proper tissue preservation was confirmed by hematoxylin–eosin staining of every 10th section.. The mounted tissue sections were deparaffinized in xylol, followed by rehydration in graded ethanol and distilled water, subsequently heated in a sodium citrate buffer (Sigma Aldrich, St. Louis, MO, USA) for 20 min at 95 °C in a water steamer, and gradually blocking buffer (ab64226, Abcam, Cambridge, UK) was administered for 30 min. The samples were incubated with primary antibodies (Table 1) overnight in a humidity chamber. The following day, they were rinsed with PBS before incubating with suitable secondary antibodies (Table 1) for one hour. The samples were then cover-slipped after being given a final PBS wash, after which the nuclei were stained with 40,6-diamidino-2-phenylindole (DAPI) (Immuno-Mount, Thermo Shandon, Pittsburgh, PA, USA). No staining was observed when primary antibodies were omitted from the immunofluorescence protocol.

Each primary antibody was diluted in blocking solution to the exact concentration before the preadsorption test was conducted. After introducing an appropriate peptide antigen, the sections were treated with the mixture. The outcomes revealed no antibody staining. When primary antibodies were left out of the immunofluorescence technique, neither non-specific secondary antibody binding nor false-positive results were seen.

### 2.4. Data Acquisition and Analysis

Sections were examined by an immunofluorescence microscope (BX51, Olympus, Tokyo, Japan) equipped with a Nikon DS-Ri2 camera (Nikon Corporation, Tokyo, Japan). In order to quantify the immunoexpression of proteins of interest, non-overlapping visual fields were captured at ×40 magnification and constant exposure times for analysis. At least ten images of the embryonic kidney structures—metanephric mesenchyme (mm), renal vesicles (rv), immature glomeruli (G), convoluted tubules (Ct), ampullae (A), and collecting ducts (Cd)—were taken at embryonic days E13.5 and E15.5 and at least twenty images of the postnatal kidney structures—glomeruli (G), proximal convoluted tubules (PCT) and distal convoluted tubules (DCT) at postnatal days P4, P11, and P14. The captured photos were then processed in ImageJ software (National Institutes of Health, Bethesda, MD, USA) and Adobe Photoshop (Adobe, San Jose, CA, USA). The cells that gave an immunoreactive signal were counted and expressed as a percentage of total cells per animal group in the previously created Excel table. Any degree of staining with the used markers in the cytoplasm, nucleus, or membrane was considered positive. The staining intensity of distinct kidney structures was semi-quantitatively evaluated at four degrees: the absence of any reactivity (−), mild reactivity (+), moderate reactivity (++), and strong reactivity (+++). Three researchers, blinded to the strain of the mice and the time points, independently evaluated the microphotographs. Interclass correlation analysis was used to test interrater agreement, and the result showed high agreement with a coefficient of >0.75 [37].

### 2.5. Statistical Analyses

GraphPad Prism 9.0.0 was used to conduct the statistical analyses (GraphPad Software, San Diego, CA, USA) with a probability level of *p* < 0.05 regarded as statistically significant. A two-way ANOVA test followed by Tukey’s multiple comparison test was used to identify significant differences in the percentage of positive cells between mm, rv/G, Ct, and A/Cd on E13.5 and E15.5 and G, PCT, and DCT at P4, P11, and P14. The mean ± standard deviation was used to express the percentage of positive cells (SD).

## 3. Results

At embryonic days E13.5 and E15.5, immunoexpression of LC3B, GRP78, HSC70, and LAMP2A were examined on metanephric mesenchyme (mm), renal vesicles (rv), convoluted tubules (Ct), ampullae (A), and collecting ducts (Cd), as well as at postnatal days P4, P11, and P14 on glomeruli (G), proximal convoluted tubules (PCT), and distal convoluted tubules (DCT) on kidneys of three animals of wild-type (wt) and *yotari* mice genotype, per for each observed time point.

### 3.1. LC3B Expression

**Figure 1:**
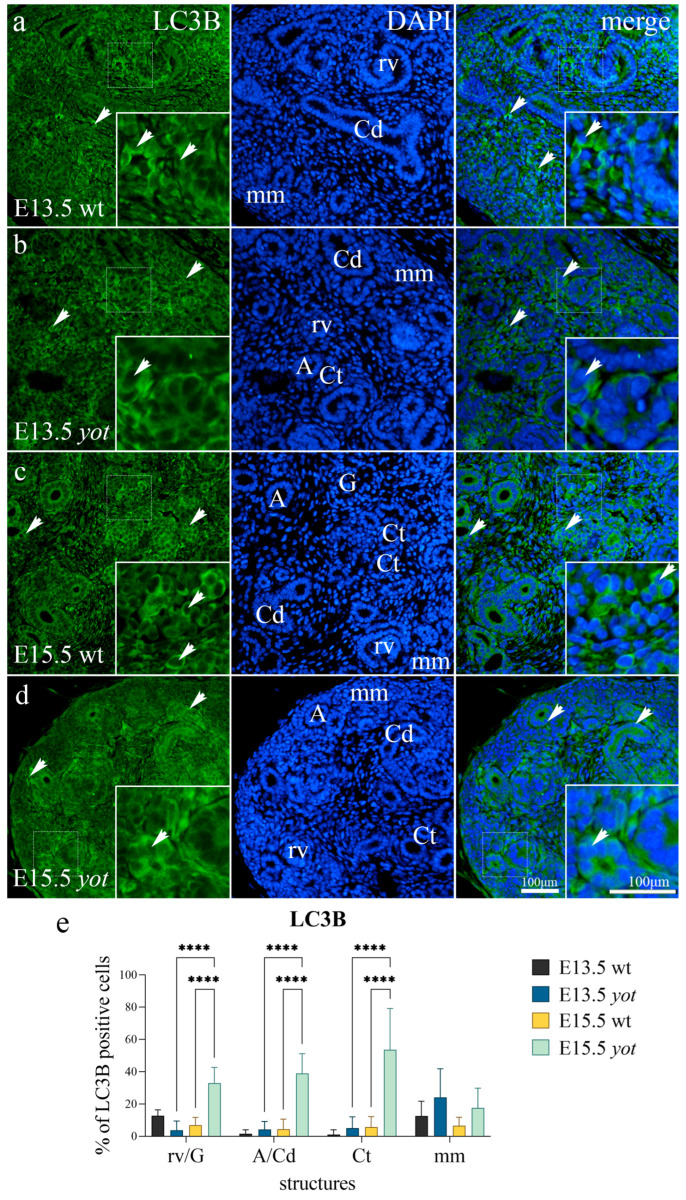
Immunofluorescence staining of developing wild-type (wt) (**a**,**c**) and *yotari* (**b**,**d**) mouse kidneys with LC3B marker and DAPI nuclear staining. Three mice were used for each genotype (*yotari* and wt) at every observed time point. Arrows show the expression pattern of LC3B in the metanephric mesenchyme (mm), renal vesicles (rv), glomeruli (G), convoluted tubules (Ct), ampullae (A), and collecting ducts (Cd) indicated on the 4′,6-diamidino-2-phenylindole (DAPI) image. The most prominent protein expression area is shown in the inserts, corresponding to the dashed boxes. Images were taken at a magnification of ×40. The scale bar is 100 μm, which refers to all images. Graph (**e**) shows the distribution of the percentage of LC3B-positive cells in the metanephric mesenchyme (mm), renal vesicles (rv) or glomeruli (G), convoluted tubules (Ct), and ampulla (A) or collecting ducts (Cd) of wild-type and *yotari* kidneys at embryonic days E13.5 and E15.5. Data are presented as the mean ± SD (vertical line) and analyzed by a two-way ANOVA test followed by Tukey’s multiple comparison test. Significant differences are indicated by **** *p* < 0.00001. At each time point, ten substructures were assessed.

At E13.5 wt, a mild diffuse signal was observed in developing nephrons (renal vesicles) but weakly in collecting ducts, including ampullae, ureteric buds, and convoluted tubules. In the surrounding undifferentiated cells of the metanephric mesenchyme (interstitium), there was a mild punctate expression of LC3B, weakening towards more differentiated kidney structures (Figure 1a). In E13.5 *yotari* mice, there was no reactivity in renal vesicles, ampullae/convoluted tubules, or collecting ducts, but LC3B signal was observed in cells of the metanephric mesenchyme, both in undifferentiated and differentiated cells (Figure 1b). Compared to the wt, the LC3B signal in mm was stronger both on the periphery and between other structures but without statistical significance (*p* = 0.661).

The signal pattern for kidneys at E15.5 wt was entirely different from that of E13.5. Visceral cells of developing glomeruli in the control group displayed weak punctate LC3B signal. In terms of other structures, convoluted tubules and ampullae/collecting ducts showed a weak punctate signal of the apical membrane. Cells in the metanephric mesenchyme surrounding the collecting ducts exhibited a predominance of LC3B expression, whereas the pattern was reversed in the periphery (Figure 1c). A much stronger LC3B signal with a somewhat different pattern was observed in E15.5 *yotari*. A strong diffuse signal of both visceral cells and the 0,0. layer of the Bowman’s capsule in glomeruli were present. The punctate signal of convoluted tubules and ampullae/collecting ducts in the wt was replaced with a strong diffuse signal of the apical membrane of the structures mentioned above. The signal in the metanephric mesenchyme did not increase (Figure 1d). The percentage of LC3B-positive cells within all observed structures, except metanephric mesenchyme, was higher in *yotari* mice (*p* < 0.05).

Semi-quantitative analysis of both animal genotypes at E13.5 revealed staining in metanephric mesenchyme, with mild intensity in wt and moderate in *yotari* (Table 2). In contrast to mild reactivity in the cells of collecting ducts and convoluted tubules/ampullae of the wt mice, *yotari* showed moderate reactivity according to semi-quantitative analysis. Furthermore, renal vesicles in mutant animals showed mild reactivity at E15.5 (Table 2).

**Figure 2:**
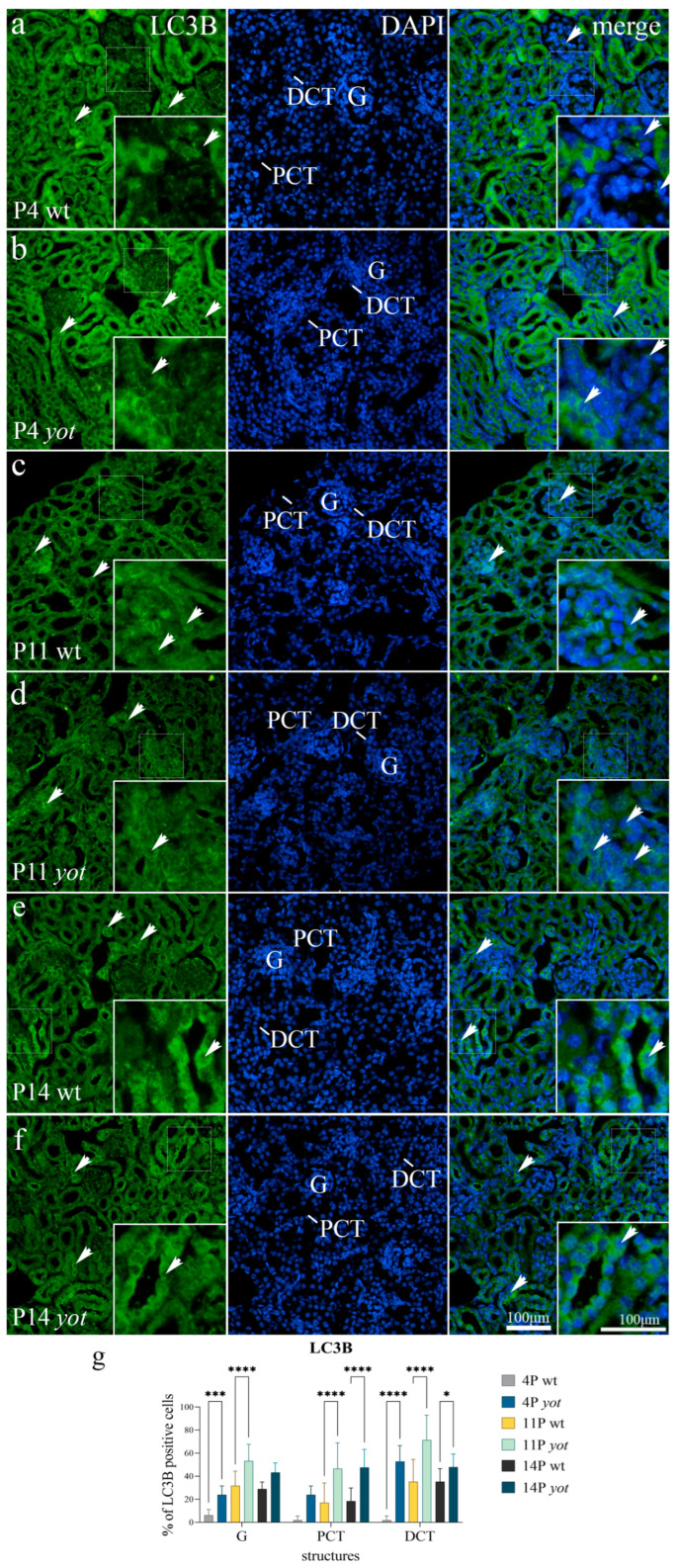
Immunofluorescence staining of postnatal wild-type (wt) (**a**,**c**,**e**) and *yotari* (**b**,**d**,**f**) mouse kidneys with LC3B marker and DAPI nuclear staining. Three mice were used for each genotype (*yotari* and wt) at every observed time point. Arrows show the expression pattern of LC3B in glomeruli (G), proximal convoluted tubules (PCT), and distal convoluted tubules (DCT) indicated on the 4′,6-diamidino-2-phenylindole (DAPI) image. The most prominent protein expression area is shown in the inserts, corresponding to the dashed boxes. Images were taken at a magnification of ×40. The scale bar is 100 μm, which refers to all images. Graph (**g**) shows the distribution of the percentage of LC3B-positive cells in the glomeruli (G), proximal convoluted tubules (PCT), and distal convoluted tubules (DCT) of postnatal kidneys of wild-type and yotari animals over time (P4, P11, P14). Data are presented as the mean ± SD (vertical line) and analyzed by a two-way ANOVA test followed by Tukey’s multiple comparison test. Significant differences are indicated by * *p* < 0.05, *** *p* < 0.0001, **** *p* < 0.00001. At each time point, twenty substructures were assessed.

When postnatal kidneys from wt genotype were analyzed, it was observed that DCT cells had a weak diffuse signal in their cytoplasm, while PCT cells had no staining in P4. The apical membrane contained the majority of this signal. Glomeruli had weak diffuse cytoplasmatic staining in the juxtaglomerular apparatus (JGA) accompanied with a weak punctate staining in the endothelial cells of capillary loops (Figure 2a). The *yotari* genotype P4 had a considerably greater percentage of LC3B-positive cells. All observed structures had substantially stronger, more diffuse signals (Figure 2b). DCT staining was observed in both the apical and basolateral membranes, whereas PCT had an apically dispersed diffuse signal (*p* < 0.0001). Glomeruli displayed a similar pattern to wt, with significantly stronger staining near JGA and blood vessel endothelial cells (*p* = 0.0004).

There was a significant increment in LC3B staining in the postnatal kidneys at later developmental stages P11 and P14. In both the aforementioned time points, *yotari* mice had a percentage of positive cells significantly higher than the control group in glomeruli (*p* < 0.05), as well as in DCT and PCT (*p* < 0.001). At P11 and P14, diffuse visceral cell glomeruli staining was seen, along with a strong punctate signal in Bowman’s capsule and JGA. In the convoluted tubules of *yotari* mice, the LC3B staining was punctate and dispersed throughout the cytoplasm. The immunoexpression pattern was similar in wt genotypes, but most cells were LC3B negative (Figure 2c–f).

Semi-quantitative analysis revealed mild reactivity in glomeruli for both animal genotypes and moderate reactivity in PCT and DCT of *yotari* mice at P4. Staining intensity overall increased in later developmental days, with mild staining in glomeruli and DCT for wt animals at P11 and moderate staining in the same structures for *yotari*. Additionally, PCT showed mild signal reactivity in mutated animals. P14 *yotari* mice displayed moderate staining intensity in all structures, in contrast to P14 wt mice, which displayed the same staining pattern as P11 wt mice (Table 3).

### 3.2. GRP78 Expression

**Figure 3:**
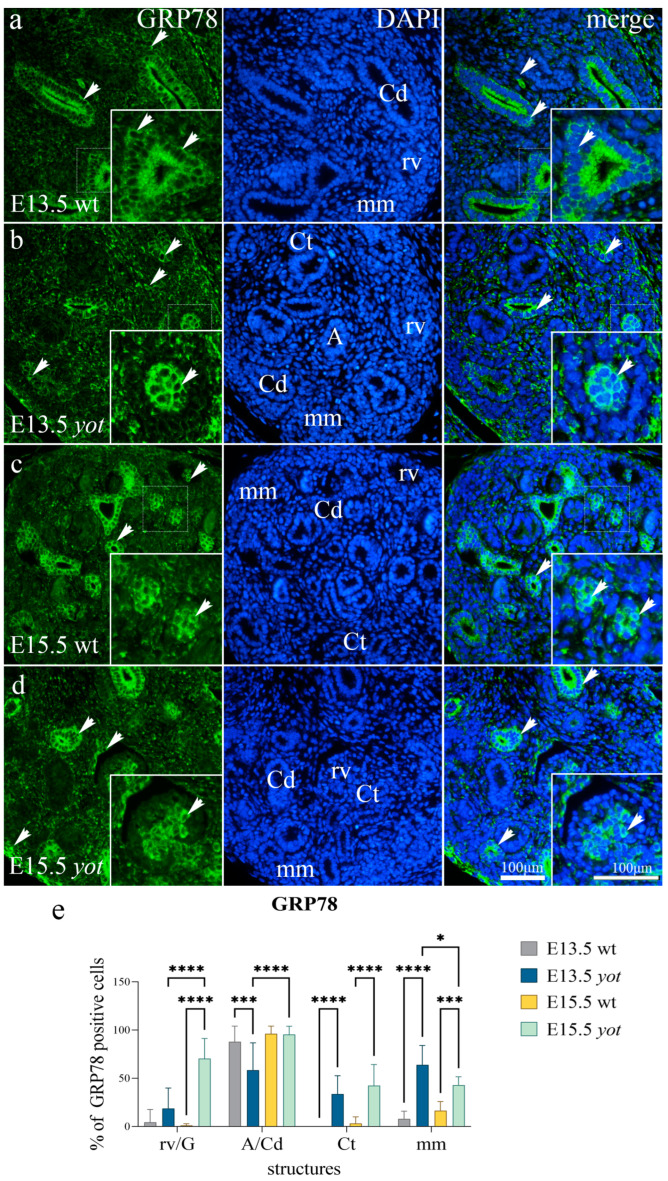
Immunofluorescence staining of developing wild-type (wt) (**a**,**c**) and *yotari* (**b**,**d**) mouse kidneys with GRP78 marker and DAPI nuclear staining. Three mice were used for each genotype (*yotari* and wt) at every observed time point. Arrows show the expression pattern of GRP78 in the metanephric mesenchyme (mm), renal vesicles (rv), glomeruli (G), convoluted tubules (Ct), ampullae (A), and collecting ducts (Cd) indicated on the 4′,6-diamidino-2-phenylindole (DAPI) image. The most prominent protein expression area is shown in the inserts, corresponding to the dashed boxes. Images were taken at a magnification of ×40. The scale bar is 100 μm, which refers to all images. Graph (**e**) shows the distribution of the percentage of GRP78-positive cells in the metanephric mesenchyme (mm), renal vesicles (rv) or glomeruli (G), convoluted tubules (Ct), and ampulla (A) or collecting ducts (Cd) of wild-type and *yotari* kidneys at embryonic days E13.5 and E15.5. Data are presented as the mean ± SD (vertical line) and analyzed by a two-way ANOVA test followed by Tukey’s multiple comparison test. Significant differences are indicated by * *p* < 0.05, *** *p* < 0.0001, **** *p* < 0.00001. At each time point, ten substructures were assessed.

Both genotypes displayed increased GRP78 expression in the collecting ducts and ampullae at E13.5. In control animals, staining was strong and diffuse throughout the cytoplasm of the aforementioned structures, while in mutated ones, the signal was mainly located in the apical membrane (Figure 3a). In *yotari* mice, a strong punctate signal was visible in metanephric mesenchyme, both in the undifferentiated and differentiating regions. Renal vesicles and collecting ducts had mild punctate staining that was mostly concentrated in the cytoplasm of cells, both apically and basolaterally (Figure 3b). There were a few statistically significant results for this time point; the control group had a higher signal intensity in a/cd (*p* = 0.0002), whereas *yotari* had a much higher intensity in ct and mm (*p <* 0.0001). At E13.5, a semi-quantitative analysis revealed strong reactivity in the wt ampullae/collecting ducts. The *yotari* exhibits mild reactivity in glomeruli and moderate reactivity for other observed structures (Table 2).

The staining pattern remained the same at E15.5, but *yotari* mice displayed substantially greater signal intensities throughout all structures (Figure 3c,d). These animals’ glomeruli stage had a much higher percentage of GRP78-positive cells than the renal vesicle stage at E13.5 (*p* < 0.0001). As for the semi-quantitative analysis, wt mice at E15.5 showed weak reactivity for all observed structures, while *yotari* structures were moderate to strongly positive (Table 2).

**Figure 4:**
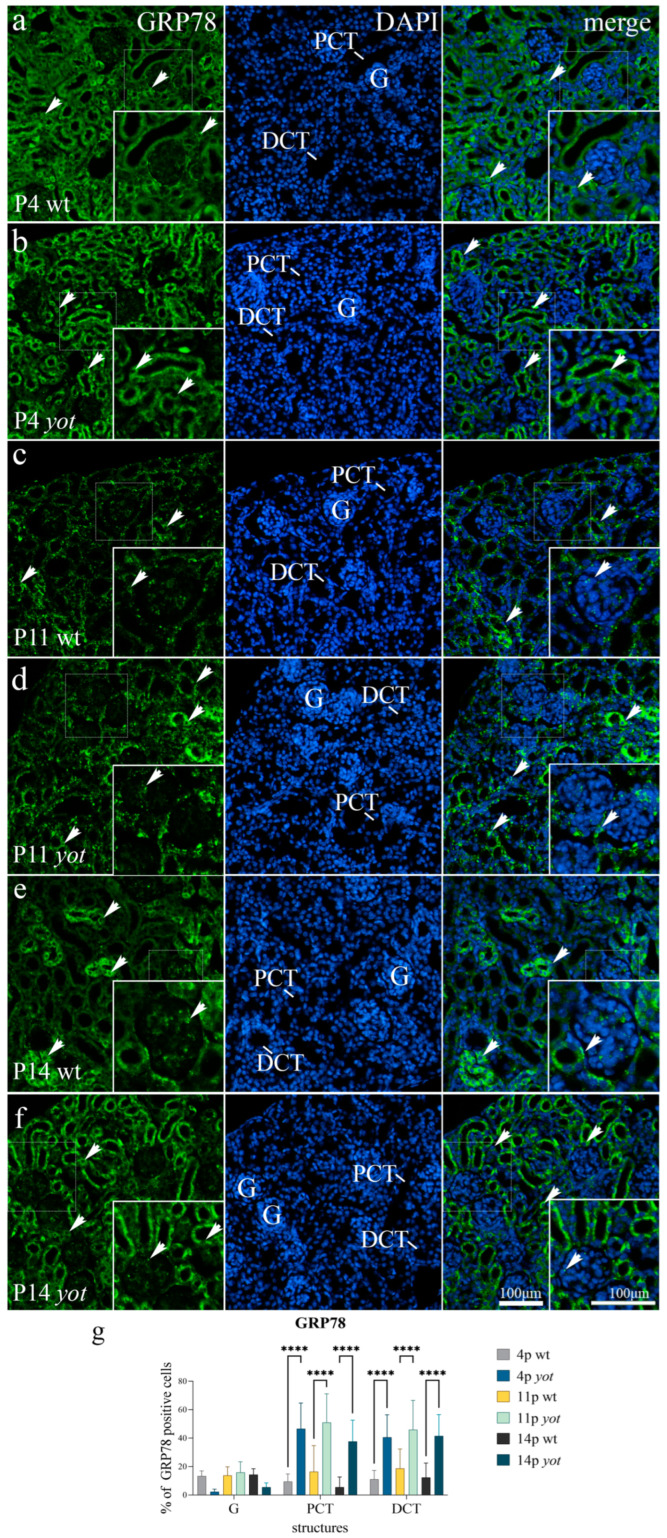
Immunofluorescence staining of postnatal wild-type (wt) (**a**,**c**,**e**) and *yotari* (**b**,**d**,**f**) mouse kidneys with GRP78 marker and DAPI nuclear staining. Three mice were used for each genotype (*yotari* and wt) at every observed time point. Arrows show the expression pattern of GRP78 in glomeruli (G), proximal convoluted tubules (PCT), and distal convoluted tubules (DCT) indicated on the 4′,6-diamidino-2-phenylindole (DAPI) image. The most prominent protein expression area is shown in the inserts, corresponding to the dashed boxes. Images were taken at a magnification of ×40. The scale bar is 1000 μm, which refers to all images. Graph (**g**) shows the distribution of the percentage of GRP78-positive cells in the glomeruli (G), proximal convoluted tubules (PCT), and distal convoluted tubules (DCT) of postnatal kidneys of wild-type and yotari animals over time (P4, P11, P14). Data are presented as the mean ± SD (vertical line) and analyzed by a two-way ANOVA test followed by Tukey’s multiple comparison test. Significant differences are indicated by **** *p* < 0.00001. At each time point, twenty substructures were assessed.

GRP78 positive cells displayed a strong diffuse signal in the postnatal stages of development, with mainly positive cells in convoluted tubules. In wt animals, immunoexpression was observed in visceral cells of the glomeruli, with the punctate signal being localized mainly within the nucleus (Figure 4a,c). In the more advanced developmental phases, the glomeruli staining became faint and almost completely absent at P14 (Figure 4e). However, expression of the positive GRP78 signal showed a completely reversed pattern in convoluted tubules, with the signal rapidly increasing throughout time (Figure 4b,d,f). *Yotari* demonstrated a significant increase in expression rate in both PCT and DCT at all observed time points, with the signal located primarily in the apical cytoplasm of these structures (*p* < 0.0001).

In the semi-quantitative evaluation, wt mice revealed mild staining in the G and PCT at P4. Same-age *yotari* specimens showed moderate intensity in cortical tubules. At P11 the control group of animals displayed mild signals for all observed structures, while *yotari* had mild intensity in G but a moderate to the strong percentage of positive cells in PCT and DCT. Lastly, G and DCT were mildly stained in P14 wt mice, while *yotari* convoluted tubules showed moderate signal intensity (Table 3).

### 3.3. HSC70 Expression

**Figure 5:**
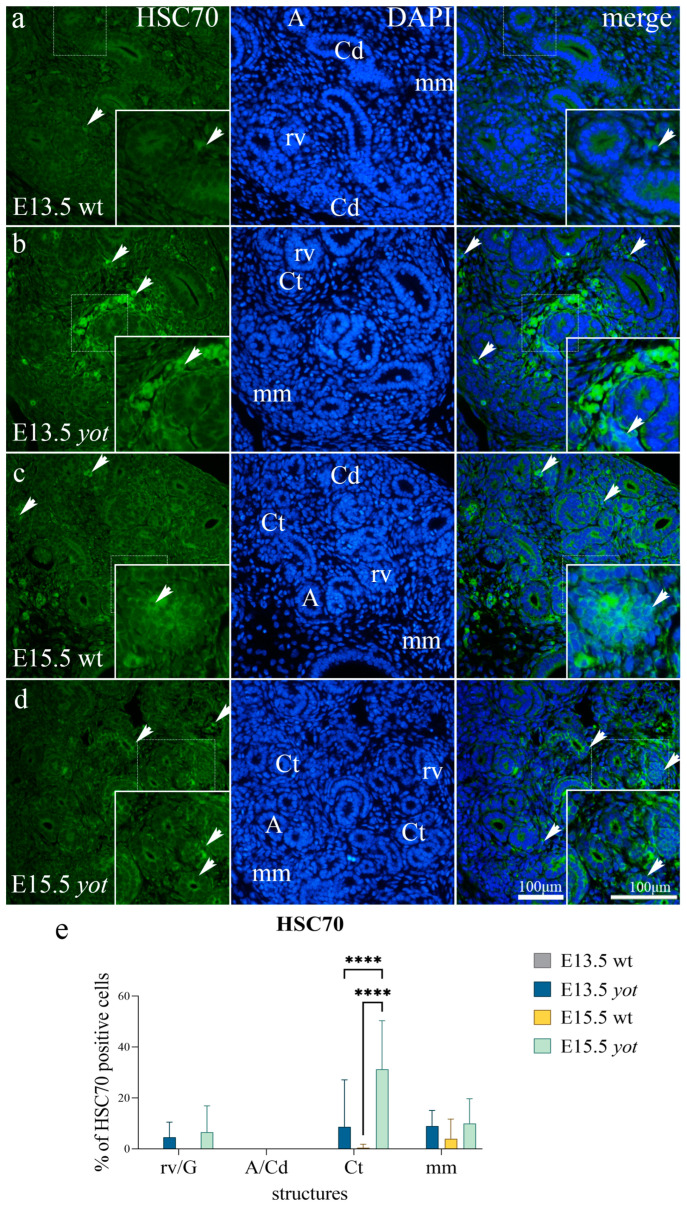
Immunofluorescence staining of developing wild-type (wt) (**a**,**c**) and *yotari* (**b**,**d**) mouse kidneys with HSC70 marker and DAPI nuclear staining. Three mice were used for each genotype (yotari and wt) at every observed time point. Arrows show the expression pattern of HSC70 in the metanephric mesenchyme (mm), renal vesicles (rv), glomeruli (G), convoluted tubules (Ct), ampullae (A), and collecting ducts (Cd) indicated on the 4′,6-diamidino-2-phenylindole (DAPI) image. The most prominent protein expression area is shown in the inserts, corresponding to the dashed boxes. Images were taken at a magnification of ×40. The scale bar is 100 μm, which refers to all images. Graph (**e**) shows the distribution of the percentage of HSC70-positive cells in the metanephric mesenchyme (mm), renal vesicles (rv) or glomeruli (G), convoluted tubules (Ct), and ampulla (A) or collecting ducts (Cd) of wild-type and *yotari* kidneys at embryonic days E13.5 and E15.5. Data are presented as the mean ± SD (vertical line) and analyzed by a two-way ANOVA test followed by Tukey’s multiple comparison test. Significant differences are indicated by **** *p* < 0.00001. At each time point, ten substructures were assessed.

In the analysis of HSC70 at the embryonic phase of development, fluorescence was completely absent in wt animals (Figure 5a–d). Both E13.5 and E15.5 *yotari* animals had weak staining of HSC70-positive cells, with mild punctate immunoexpression in the cytoplasm of convoluted tubule cells and metanephric mesenchyme. The signal in Ct for *yotari* mice showed the same pattern for both embryonic time points, but the statistical difference was significant only for older specimens (*p* < 0.0001). Even though there was some punctate signal in the interstitium between differentiating structures, there was no significant difference between wt and *yotari* in the embryonic developmental phases (*p* = 0.064).

Semi-quantitative analysis of *yotari* on E15.5 showed mild to moderate reactivity in mm and Ct (Table 2).

**Figure 6:**
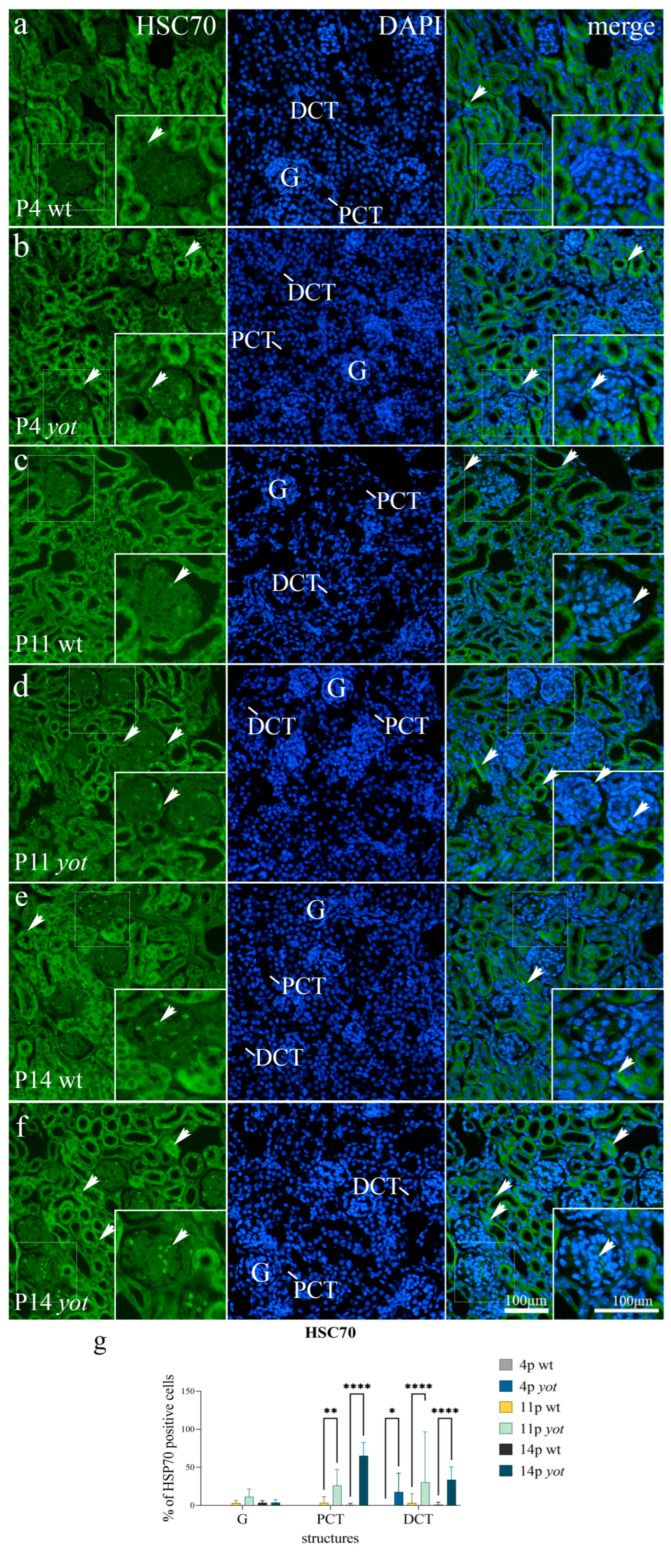
Immunofluorescence staining of postnatal wild-type (wt) (**a**,**c**,**e**) and *yotari* (**b**,**d**,**f**) mouse kidneys with HSC70 marker and DAPI nuclear staining. Three mice were used for each genotype (*yotari* and wt) at every observed time point. Arrows show the expression pattern of HSC70 in glomeruli (G), proximal convoluted tubules (PCT), and distal convoluted tubules (DCT) indicated on the 4′,6-diamidino-2-phenylindole (DAPI) image. The most prominent protein expression area is shown in the inserts, corresponding to the dashed boxes. Images were taken at a magnification of ×40. The scale bar is 100 μm, which refers to all images. Graph (**g**) shows the distribution of the percentage of HSC70-positive cells in the glomeruli (G), proximal convoluted tubules (PCT), and distal convoluted tubules (DCT) of postnatal kidneys of wild-type and yotari animals over time (P4, P11, P14). Data are presented as the mean ± SD (vertical line) and analyzed by a two-way ANOVA test followed by Tukey’s multiple comparison test. Significant differences are indicated by * *p* < 0.05, ** *p* < 0.001, **** *p* < 0.00001. At each time point, twenty substructures were assessed.

Concerning postnatal stages, the same pattern of staining continued for wt mice. The signal was completely absent; there were a few sporadic positive HSC70 cells, but the overall percentage was negligible (Figure 6a,c,e). However, the pattern was somewhat different for *yotari*. The percentage of positive HSC70 cells gradually increased over time, with the strongest signal present in cortical tubules. At P4, the signal was diffuse and mostly concentrated in the apical membrane of DCT (Figure 6b). As mice grew, so did the HSC70 signal; at P11, the percentage of positive cells increased to around 30% in the tubules of all examined animals (Figure 6d). In PCT, the signal was dispersed throughout the cytoplasm, and in DCT, it was still predominantly found in the apical membrane. At P11, the glomeruli collected some fluorescence, mostly perinuclear in visceral cells. On the 14th day of postnatal development, HSC70 staining reached its peak. Around 60% of PCT and DCT cells were positive, with the same pattern of signal distribution as P11 *yotari* (Figure 6f). As for the statistical analysis, there was no significant difference in glomeruli staining between the control and *yotari* groups of animals in any postnatal phase. However, there were significant results regarding other structures, such as PCT at P11 (*p* = 0.0023) and P14 (*p* < 0.0001), as well as DCT at P4 (*p* = 0.035), P11 (*p* < 0.0001) and P14 (*p* < 0.0001).

Based on the semi-quantitative analysis, wt animals displayed minimal PCT reactivity on the 14th postnatal day. *Yotari* samples exhibited a more complicated pattern of reactivity; semi-quantitative analysis for P4 *yotari* revealed a moderate intensity for both PCT and DCT; at P11, the signal sample remained unchanged for PCT and DCT, but at this time point, there was also minor reactivity in glomeruli cells. *Yotari* P14 exhibited strong reactivity in PCT and mild in G and DCT (Table 3).

### 3.4. LAMP2A Expression

**Figure 7:**
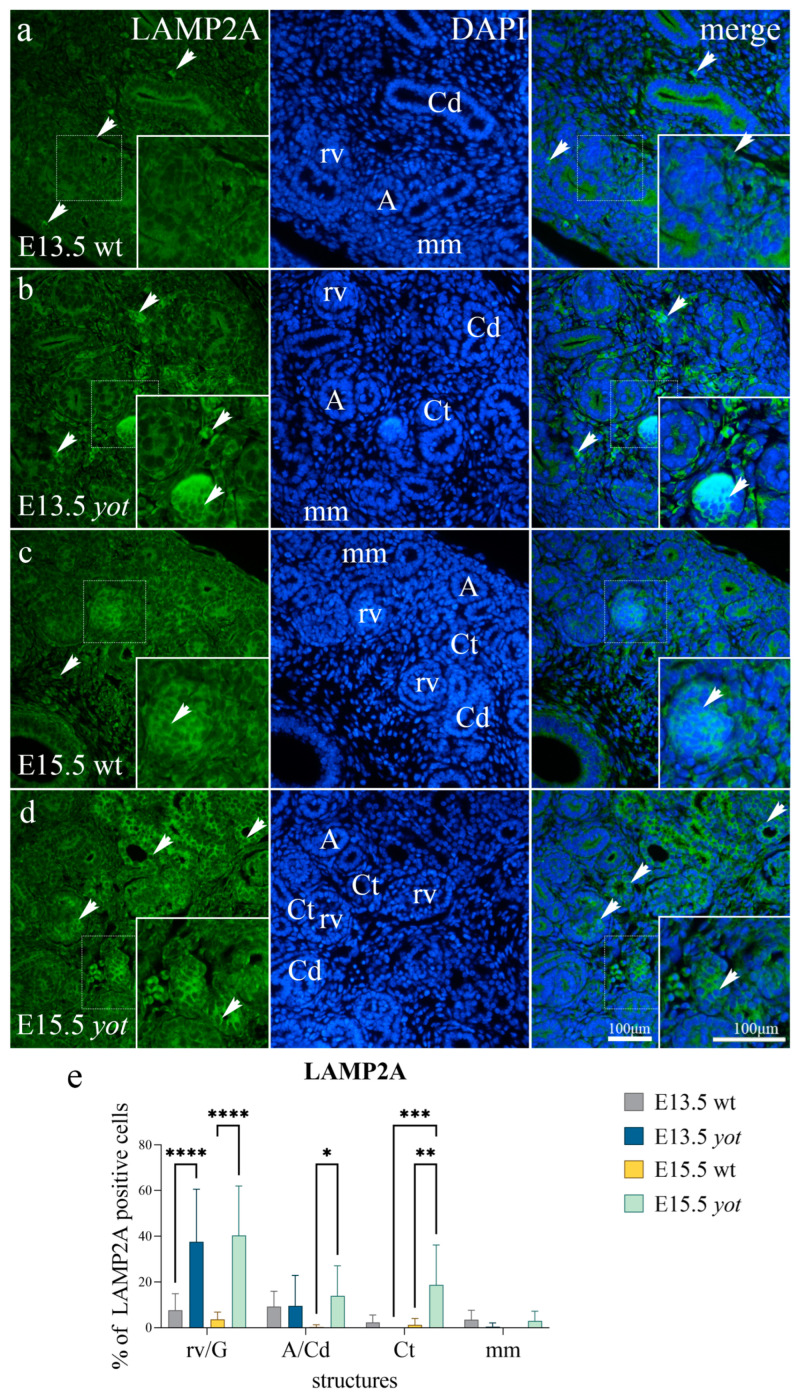
Immunofluorescence staining of developing wild-type (wt) (**a**,**c**) and *yotari* (**b**,**d**) mouse kidneys with LAMP2A marker and DAPI nuclear staining. Three mice were used for each genotype (*yotari* and wt) at every observed time point. Arrows show the expression pattern of LAMP2A in the metanephric mesenchyme (mm), renal vesicles (rv), glomeruli (G), convoluted tubules (Ct), ampullae (A), and collecting ducts (Cd) indicated on the 4′,6-diamidino-2-phenylindole (DAPI) image. The most prominent protein expression area is shown in the inserts, corresponding to the dashed boxes. Images were taken at a magnification of ×40. The scale bar is 100 μm, which refers to all images. Graph (**e**) shows the distribution of the percentage of LAMP2A-positive cells in the metanephric mesenchyme (mm), renal vesicles (rv) or glomeruli (G), convoluted tubules (Ct), and ampulla (A) or collecting ducts (Cd) of wild-type and *yotari* kidneys at embryonic days E13.5 and E15.5. Data are presented as the mean ± SD (vertical line) and analyzed by a two-way ANOVA test followed by Tukey’s multiple comparison test. Significant differences are indicated by * *p* < 0.05, ** *p* < 0.001, *** *p* < 0.0001, **** *p* < 0.00001. At each time point, ten substructures were assessed.

LAMP2A-positive cells were identified as green staining in both wt and *yotari* mice embryonic stages. At E13.5, collecting ducts and ampullae of both genotypes showed a weak fluorescence intensity, with staining concentrating primarily on the apical membrane of the aforementioned structures (Figure 7a,b). Renal vesicles of *yotari* specimens displayed stronger reactivity at E13.5, with staining concentrated perinuclearly (*p* < 0.0001).

For wt at E15.5, the pattern of reactivity remained the same (Figure 7c), while in the *yotari* genotype, the proportion of LAMP2A-positive cells varied considerably between E13.5 and E15.5 (Figure 7d). Perinuclearly in immature glomeruli, a large diffuse signal was still present, but convoluted tubules showed the biggest variation. The cytoplasm of about half of the examined ct had strong staining that was at times punctate and at other times diffuse. Statistical analysis revealed significant differences between the two genotypes: the LAMP2A expression in rv/G, A/Cd and Ct was greater at E15.5 *yotari* than in wt of the same age (*p* < 0.0001).

Semi-quantitative analysis of wt on E13.5 revealed mild staining intensity in immature glomeruli and convoluted tubules. Same-age *yotari* specimens displayed moderate staining intensity in renal vesicles. At E15.5, the wild-type displayed mild staining reactivity in rv/G and a/cd, while *yotari* specimens had the same reactivity in rv/G and a/cd but also displayed moderate intensity in ct (Table 2).

**Figure 8:**
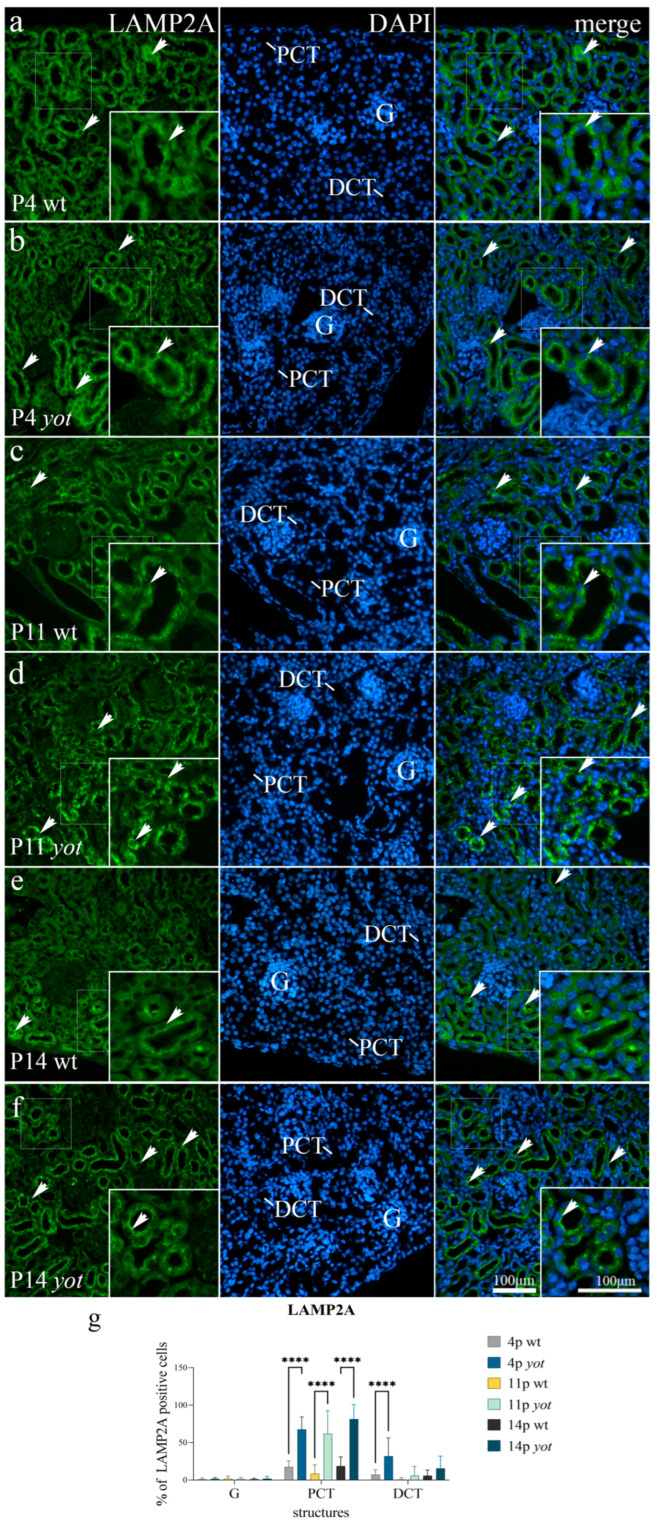
Immunofluorescence staining of postnatal wild-type (wt) (**a**,**c**,**e**) and *yotari* (**b**,**d**,**f**) mouse kidneys with LAMP2A marker and DAPI nuclear staining. Three mice were used for each genotype (*yotari* and wt) at every observed time point. Arrows show the expression pattern of LAMP2A in glomeruli (G), proximal convoluted tubules (PCT), and distal convoluted tubules (DCT) indicated on the 4′,6-diamidino-2-phenylindole (DAPI) image. The most prominent protein expression area is shown in the inserts, corresponding to the dashed boxes. Images were taken at a magnification of ×40. The scale bar is 100 μm, which refers to all images. Graph (**g**) shows the distribution of the percentage of LAMP2A-positive cells in the glomeruli (G), proximal convoluted tubules (PCT), and distal convoluted tubules (DCT) of postnatal kidneys of wild-type and yotari animals over time (P4, P11, P14). Data are presented as the mean ± SD (vertical line) and analyzed by a two-way ANOVA test followed by Tukey’s multiple comparison test. Significant differences are indicated by **** *p* < 0.00001. At each time point, twenty substructures were assessed.

The same expression pattern continued in the early postnatal stage. At P4, both genotypes expressed staining in convoluted tubules (Figure 8a,b), but the intensity in *yotari* specimens was more robust both in DCT and PCT (*p* < 0.0001). Almost 60% of all observed convoluted tubules displayed a strong, diffuse signal, predominantly concentrated in the apical membranes of *yotari animals*. The same pattern was noticed in wt animals but in a much lesser percentage.

A similar pattern was present at later postnatal stages (P11 and P14), with a smaller percentage of LAMP2A-positive cells in distal convoluted tubules. Throughout postnatal development, the signal redistribution in apical membranes of convoluted tubules remained unaltered (Figure 8c–f). A significant difference was present for PCT (*p* < 0.0001).

Semi-quantitative analysis revealed mild PCT staining for wt at P4; for the same-age *yotari*, there was mild DCT staining and strong PCT staining. *Yotari* displayed a moderate PCT signal intensity at P11, which remained constant at P14 for the same genotype, whereas wt displayed mild PCT reactivity at P14 (Table 3).

## 4. Discussion

Kidney development, combined with nephron maturing, is one of the most precisely organized and integrated processes, coordinated through the interaction of different genes and cellular mechanisms. Interference with this highly complicated multi-stage process may lead to errors in nephrogenesis, resulting in different structural defects of the kidneys and outflow tracts referred to as CAKUT. These malformations are the most common congenital disabilities and the leading cause of end-stage renal disease in children [38,39]. In our earlier research with *yotari* mice, we discovered a series of different markers and how disruption in one step of their signaling pathways can result in CAKUT [11,40,41].

We discovered the most about the genetic elements that affect CAKUT development using mouse models and are getting closer to understanding their etiology with each new gene uncovered [6]. As our previous research showed strong expression of DAB1 during fetal kidney development and diminishing that same expression in postnatal healthy kidneys [10], in this study, we assumed that *Dab1* knockout mice would have a different pattern in autophagy and apoptosis, as these two mechanisms are extremely important for normal kidney function and development [42,43]. Using a *Dab1^−/−^* mouse model, we explored various LC3B, GRP78, HSC70, and LAMP2A expression patterns during both embryonic and postnatal developmental periods to confirm our hypothesis.

This study showed no significant LC3B immunoexpression in the early stages of embryonic development at E13.5. The staining signal was mild for both genotypes, suggesting that autolysosomes were not yet formed and, consequently, autophagy was not yet initiated. However, embryos obtained at E15.5 showed a significant increase of positive LC3B cells in all observed structures except the metanephric mesenchyme. The same immunoexpression pattern carried on into the postnatal period, where *yotari* had a significantly higher percentage of LC3B-positive cells than control animals in all observed structures. In our recent study, which examined postnatal *yotari* specimens, higher LC3B expression was observed in the glomeruli of P11 and P14 mice. These findings probably reflect higher autophagy activity in podocytes, which could be associated with foot process effacement and could lead to nephrotic syndrome development [11]. However, it has been reported that inhibition of autophagy may lead to the activation of pro-apoptotic pathways of endoplasmatic reticulum stress, which induces podocyte apoptosis and could be a possible explanation for autophagy’s protective role [44,45]. Several studies have shown that in most renal diseases, autophagy largely serves a preventive function. Its activity is essential in preventing the onset of podocytopathies, especially minimal change disease (MCD), a glomerular condition linked to the nephrotic syndrome that can result in chronic renal failure [46]. In addition, downregulation of autophagy, such as in blood hyperglycemia, can cause diabetic kidney disease [47], leading to fibrosis in renal epithelial cells, including podocytes, proximal tubular, as well as in mesangial, and endothelial cells. The nephrotoxin and chemotherapeutic agent cisplatin elevates LC3B expression in proximal tubular cells. The acute kidney injury and renal dysfunction caused by cisplatin were significantly exacerbated by autophagy inhibitors (such as the antimalarial drug chloroquine) [48]. Most studies mentioned above suggest an entirely cytoprotective role for autophagy in acute kidney injuries and chronic kidney diseases through recycling damaged cellular components, making its pathway a potential therapeutic target. Still, this process can also be detrimental in certain conditions, and further rigorous investigations are required.

Both genotypes expressed a high percentage of GRP78-positive cells at embryonic stages, with *yotari* having a stronger signal in convoluted tubules and metanephric mesenchyme. At E15.5, *yotari* exhibits elevated expression proportions in the cytoplasm of maturing glomeruli cells. In the postnatal period, *yotari* has a significantly higher percentage of positive cells in both PCT and DCT for every observed period. This immunoexpression pattern suggests an essential role for GRP78 in the early embryonic maturation of ampullae and collecting ducts but also signalizes significant levels of ER stress in all of the examined postnatal *yotari* structures. GRP78, a well-known chaperone engaged in numerous intracellular processes and a sensor for ER stress, prevents programmed cell death by promoting protein folding and eliminating misfolded proteins. However, Runbing et al. suggest that it may also serve an opposite function by promoting renal tubular fibrosis via the IRE1/XBP1 signaling pathway [24,49,50]. Earlier studies using *GRP78* knockout mice provided direct evidence that GRP78 is essential for early embryo development and that its absence could lead to a series of pro-apoptotic pathways and early embryonic lethality [51,52], thus explaining the high percentage of GRP78-positive cells during embryonic stages in wt mice. Although there are currently no studies comparing the immunoexpression of Dab1 and GRP78 proteins in kidneys, a study by Mimura et al. showed that in knock-in *GRP78* mice, higher expression of dephosphorylated Dab1 in the brain was observed, with reduced expression of Reelin, an extracellular matrix glycoprotein upstream of Dab1. This study suggested that GRP78 is essential for proper brain development and adequate Reelin/Dab1 pathway regulation [53]. During ER stress, multiple so-called stress proteins dissociate from GRP78 and then activate the downstream signaling pathway to promote the degradation of unfolded or misfolded proteins. GRP78 staining in *yotari* postnatal convoluted tubules could thus indicate induction of ER stress, as has already been described in most acute and chronic renal pathologies [23,54,55]. These findings suggest that functional *Dab1* gene silencing may cause an accumulation of unfolded or misfolded proteins in the ER lumen, thereby upregulating the GRP78 pathway and, as a result of autophagy capacity oversaturation, making apoptosis hypothetically the central mechanism of renal dysfunction in *yotari* mice.

The significant difference at embryonic stages in the percentage of HSC70-positive cells was found at E15.5, where *yotari* exhibited an increase in convoluted tubules. The same pattern was observed in the postnatal developmental stages analysis. *Yotari* mice expressed higher signal intensity at every timepoint except for P4, where there was not a significant difference in the reactivity of PCT cells. Chaperone-mediated autophagy (CMA) is a selective protein degradation process precisely mediated by HSC70. In the study of Cuervo et al., it is demonstrated that levels of α2-microglobulin (CMA substrate) in kidneys and liver after exposure to 2,2,4-trimethylpentane are increased, thus inducing the lysosome overload and resulting in severe cellular damage [56]. During acute diabetes mellitus, CMA was significantly inhibited in the rat renal cortex, and a mass of proteins with a KFERQ-like motif increased [57]. Unfortunately, no research has demonstrated a connection between the Reelin/Dab1 pathway and CMA mediators such as HSC70. However, based on the previous results, we can infer that in *yotari* mice, a higher percentage of HSC70-positive cells correlates with increased CMA activity and plays a protective role in cortical tubules.

During gestation, LAMP2A immunoexpression was observed in immature glomeruli, where *yotari* mice exhibit a significantly higher percentage of LAMP2A-positive cells than wt mice. The convoluted tubule LAMP2A staining pattern resembled HSC70 immunoexpression, which was significantly higher in the later stages of *yotari* mouse embryonic development. While PCT showed a significant difference in all examined postnatal periods compared to control animals, DCT of the *yotari* mice had a higher percentage of positive cells only in P4. It was predicted that LAMP2A’s staining pattern would be similar to that of HSC70 since LAMP2A serves as a receptor for chaperones carrying unfolded or misfolded proteins. The only study explaining the localization of LAMP2A in mutated kidneys is by Zhang et al. They discovered the apical distribution of LAMP2A in the cystinosis-relevant kidney PCT but its absence in the basal areas of the proximal tubule cells using *Ctns^−/−^* mice. These findings could implicate that absence of Dab1 results in the accumulation of CMA substrates in proximal tubule cells, which consequently leads to higher expression of the lysosomal receptor LAMP2A [20,58]. Recent research using *LAMP2A* knockout mice showed that cells are more vulnerable to stresses when this receptor is absent. Earlier work has demonstrated the significance of LAMP2A as a CMA regulator, demonstrating how CMA activity to eliminate proteins oxidized during mild oxidative stress appears to occur through increased *LAMP2A* transcription [59,60].

The dynamic and expression patterns of LC3B, GRP78, HSC70, and LAMP2A in control and *yotari* animals demonstrate the significance of these markers both during kidney development and later on in maintaining proper renal function. We can assume that silencing the *Dab1* gene causes an accumulation of unfolded or misfolded proteins, leading to increased expression of chaperones and other autophagic biomarkers. Different stimuli control CMA activity, including hunger, growth hormones, oxidative stress, lipids, aging, and retinoic acid signaling. The exact mechanism that initiates the autophagic sequence in *Dab1^−/−^* mice remains unknown but based on the increased percentage of GRP78-positive cells, we can assume that oxidative stress plays a significant role, particularly in convoluted tubules in both embryonic and adult kidneys.

In conclusion, our research emphasizes the importance of autophagy in kidney dysfunction and suggests that it plays a protective role in preventing programmed cell death. All observed proteins play significant roles in the autophagy process, making them excellent biomarkers for quantifying it. For various renal diseases, modifying autophagic activity may be a promising therapeutic approach; however, more studies are required to fully understand the role of autophagy in *Dab1*^−/−^ kidney hypoplasia.

## Figures and Tables

**Table 1:** Antibodies used for immunofluorescence.

Antibodies		Host	Dilution	Source
Primary	Anti-LC3B/ab48394	Rabbit	1:100	Abcam (Cambridge, UK)
	Anti-GRP78/PA5-19503	Rabbit	1:300	Thermo Fisher Scientific (Waltham, MA, USA)
	Ani-HSC70/ab51052	Rabbit	1:50	Abcam (Cambridge, UK)
	Anti-LAMP2A/ab18528	Rabbit	1:100	Abcam (Cambridge, UK)
Secondary	Anti-Rabbit IgG, Alexa Fluor^®^ 488, 711-545-152	Donkey	1:300	Jackson Immuno Research Laboratories, Inc. (Baltimore, PA, USA)

**Table 2:** Staining intensity of specific antibodies in the kidneys of *yotari* and wild-type mice at embryonic days E13.5 and E15.5.

Embryonic Day (E)	Animal	Structure	Antibody			
			LC3B	GRP78	HSC70	LAMP2A
E13.5	wild-type	mm	+	−	−	−
		rv/G	+	−	−	+
		Ct	−	−	−	+
		A/Cd	−	+++	−	−
	*yotari*	mm	++	++	+	−
		rv/G	−	+	−	++
		Ct	−	++	−/+	−
		A/Cd	−	++	−/+	−
E15.5	wild-type	mm	−/+	−/+	−/+	−
		rv/G	−/+	−	−	+
		Ct	+	−	−	−
		A/Cd	+	+++	−	+
	*yotari*	mm	−/+	++	+	−
		rv/G	+	++	−/+	+
		Ct	++	++	++	++
		A/Cd	++	+++	−	+

+++ strong reactivity; ++ moderate reactivity; + mild reactivity; − no reactivity; mm—metanephric mesenchyme, rv—renal vesicle, G—immature glomeruli, Ct—convoluted tubule, A—ampulla, Cd—collecting duct, E—day of embryonic development; 1B-light chain 3 (LC3B), glucose-regulated protein 78 (GRP78), heat shock cognate 71-kDa protein (HSC70), and lysosomal-associated membrane protein 2A (LAMP2A).

**Table 3:** Staining intensity of specific antibodies in the kidneys of *yotari* and wild-type mice at postnatal days P4, P11, and P14.

Postnatal Day (P)	Animal	Structure	Antibody			
			LC3B	GRP78	HSC70	LAMP2A
P4	wild-type	G	+	+	−	−
		PCT	−	+	−	+
		DCT	−/+	−/+	−	−
	*yotari*	G	+	−	−	−
		PCT	++	++	−	+++
		DCT	++	++	+	+
P11	wild-type	G	+	+	−/+	−/+
		PCT	−/+	+	−/+	−/+
		DCT	+	+	−/+	−
	*yotari*	G	+++	+	+	−/+
		PCT	+	+++	++	++
		DCT	+++	++	++	−
P14	wild-type	G	+	+	−	−/+
		PCT	−	−	+	+
		DCT	+	+	−	−/+
	*yotari*	G	++	−/+	+	−
		PCT	++	++	+++	+++
		DCT	++	++	+	+

+++ strong reactivity; ++ moderate reactivity; + mild reactivity; − no reactivity; G—glomeruli, PCT—proximal convoluted tubules, DCT—distal convoluted tubules, P—day of postnatal development.

## Data Availability

All data and materials are available upon request.
